# Preface: Computational and experimental methods for biological research: cardiovascular diseases and beyond

**DOI:** 10.1186/s12938-016-0269-8

**Published:** 2016-12-28

**Authors:** Dalin Tang, Zhi-Yong Li

**Affiliations:** 1 School of Biological Science and Medical Engineering, Southeast University, Nanjing, 210096 China; 2 Department of Mathematical Sciences, Worcester Polytechnic Institute, Worcester, MA 01609 USA

Cardiovascular disease (CVD) is the leading cause of death worldwide. Huge effort has been made in many disciplines including medical imaging, computational modeling, biomechanics, bioengineering, medical devices, animal and clinical studies, population studies as well as genomic, molecular, cellular and organ-level studies seeking improved methods for early detection, diagnosis, prevention and treatment of these diseases. Following the success of our special issue last year [1], experts in various disciplines were invited to write papers covering important areas in biomedical research, including medical images [2–5], arteries [6–10], aneurysm [11–14], some heart and lung issues [15–17], medical devices and treatment techniques [18–24], microscale studies at cell and molecule levels [25–28], and others [29–36]. A total of 35 papers were included in this special issue, with a good spectrum of coverage.

In the modern diagnosis process, medical images have the utmost importance. Zhu et al. presented a feasibility study of T2-prepared segmented 3D-Gradient-Echo for fast T2-weighted high-resolution three-dimensional imaging of the carotid artery wall at 3T [2]. They reported that the acquisition time using 3D-Gradient-Echo could be substantially reduced to about 25% of the respective 3D-TSE technique. Xing et al. [3] proposed a new protocol for computing wall shear stress based on contrast-enhanced micro-CT imaging in murine carotid arteries. Contrast-enhanced micro-CT was performed using eXIA 160. They reported that eXIA 160-enhanced micro-CT allowed clear visualization and assessment of the RCCA in all eight animals. No adverse biological effects were observed from the use of eXIA 160. Li et al. [4] used machine learning algorithm models in ApoE−/− mice to predict carotid plaque progression. They found that contralateral carotid artery diameter at 7 days after surgery was the most reliable predictive factor in plaque progression. They achieved over 87.5% accuracy, 80% sensitivity, and 95% specificity with support vector machine (SVM). Tian et al. [5] provided comparison of lesion outline and temperature field determined by different ways in atrial radiofrequency ablation.

In the “artery” category, we have papers covering deformation of three-dimensional red blood cells in non-uniform capillaries [6], study of neo-aortic root for arterial switch operation [7], study of effect of LVAD on aortic blood flow pattern [8], and study for the risk of Stanford type-A aortic dissection with different tear size and location [9]. It also included a study of mechanical anisotropy of porcine thoracic aorta by uniaxial tensile tests [10].

For aneurysm, Xiong et al. [11] presented a hemodynamics study of an innovative multilayer stent for treatment of aneurysms. Li et al. [12] studied the pressure shielding ability of stent-graft after endovascular aneurysm repair (EVAR) of abdominal aortic aneurysm (AAA). Zhang introduced a phantom-based experimental validation of fast virtual deployment of self-expandable stents for cerebral aneurysms [13]. Xu and Liu studied potential association between flow instability and rupture in patients with matched-pairs of ruptured-unruptured intracranial aneurysms [14]. Their results demonstrated highly disturbed states of the blood flows in the ruptured aneurysms of the two patients with multiple aneurysms. The ruptured aneurysms exhibit obviously temporal intra-cycle wall shear stress (WSS) fluctuations rather than the unruptured aneurysms of the same patient. Cycle-to-cycle fluctuations are further observed in the ruptured aneurysms when the flow turns to decelerate [14].

Effectiveness of stents and grafts were studied in [18, 22, 23]. Gu et al. [21] studied the effect of captopril on the performance of the control strategies of BJUT-II VAD. Zhang et al. [19] provided an optimization study of patient-specific design of flow diverters made from helix-like wires.

Micro-scale studies included β1 integrin signaling in asymmetric migration of keratinocytes under mechanical stretch in a co-cultured wound repair model [25] and mechanical regulation of calcium signaling of HL-60 on P-selectin under flow, among others. Wang et al. [28] used a multi-component parallel-plate flow chamber system for studying the effect of exercise-induced shear stress on endothelial cells. Their cellular experiments demonstrated that the actin microfilaments and the production of NO within cells exposed to the two different wall shear stress waveforms exhibit different dynamic behaviors; there are larger numbers of actin microfilaments and higher level NO in cells exposed in exercise-induced wall shear stress condition than resting wall shear stress condition. Some recent developments in computational and experimental methods for biological research beyond cardiovascular system were also included in the issue [29–36]. They included studies for bones [29, 31], mental stress [30], eyes [32–34], and tumour growth [35]. Effects of swimming training on carotid arterial stiffness and hemodynamics in young overweight adults were investigated [36]. This enabled a good coverage of different aspects of recent developments in modern methods in biological research.
